# The Natural Killer Cell Line NK-92 and Its Genetic Variants: Impact on NK Cell Research and Cancer Immunotherapy

**DOI:** 10.3390/cancers17121968

**Published:** 2025-06-13

**Authors:** Hans Klingemann

**Affiliations:** ImmunityScience LLC, 50 Liberty Drive, Boston, MA 02210, USA; hans.klingemann@gmail.com

**Keywords:** NK-92, cancer, immunotherapy, cellular therapy

## Abstract

The NK-92 cell line has become a very relevant tool for natural killer (NK) cell research, largely because it largely mirrors the characteristics of human blood-derived NK cells. It also has a doubling time of less than 30 h, making it possible to generate a significant number of cells in a relatively short time. Its safety as an anti-cancer cell therapy has been documented in over 200 cancer patients. Various genetically engineered variants have been generated that express a high-affinity Fc-receptor and various chimeric antigen receptors (CARs) and secrete immune-active cytokines. NK-92 cells expressing CARs for HER-2, PD-L1, and CD19 CAR are in advanced clinical trials in cancer patients. These cells also have cytotoxic activity against targets infected with bacteria, fungi, and viruses. More recently, the cellular lysate of NK-92 cells, generated by simple freeze/thaw, has shown anti-cancer potential when injected intra-tumor. Since a comprehensive review of NK-92 was recently published on the occasion of its 30-year “anniversary”, this review will focus on more recent research initiatives and results with the cell line.

## Simple Summary

The NK-92 cell line has functional characteristics of blood NK cells but with broader and higher cytotoxicity. It has a short doubling time and can be expansded to large numbers under FDA guided conditions. The unmodified NK-92 cells have completed several clinical trials confirming no significant side effects and clinical responses. Its CAR engineered variats are in clinical trials. New research results also confirm their anti-microbial efficacy and their cellular lysate has local and systemic anti-tumor effects.

## 1. Brief History of the Origin and Development of NK-92

**Figure 1:**
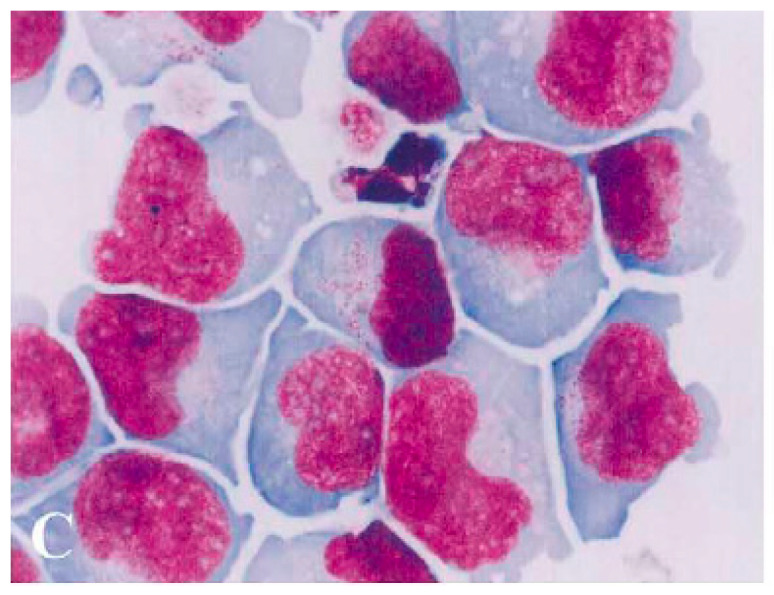
NK cells from the blood smear of a patient with NK-cell lymphoma whose cells were harvested and used to establish the NK-92 cell line. *Cover of BLOOD Febr 1st*, *1997*, *Vol*. *89*.

Back in 1992, I was called to see a patient with an unusual lymphoma at the British Columbia Cancer Agency in Vancouver/CDN. The patient’s blood and bone marrow showed morphologically classical large granular natural killer (NK) lymphocytes [Figure 1]. The cells—on flow cytometry—were CD56-positive and CD3-negative, expressing high levels of ICAM-1 and LFA-1 adhesion molecules, but were negative for the Fc-receptor [1,2]. My lab established a cell line from the patient’s blood NK cells, and since it was the year 1992, the cell line was named “NK-92”. Since its maintenance and expansion requires IL-2, the parental cell line was subsequently genetically modified to produce and secrete its own IL-2 at *low* concentrations, *NK-92ci*, or at *higher* concentrations, *NK-92mi* [3]. The cell lines were deposited with the American Type Culture Collection (ATCC, Manassas, VA, USA) and the German Collection of Microorganisms and Cell Cultures (DSMZ, Braunschweig, Germany). Researchers worldwide have had access to these cell lines since the mid-1990s.

**Figure 2:**
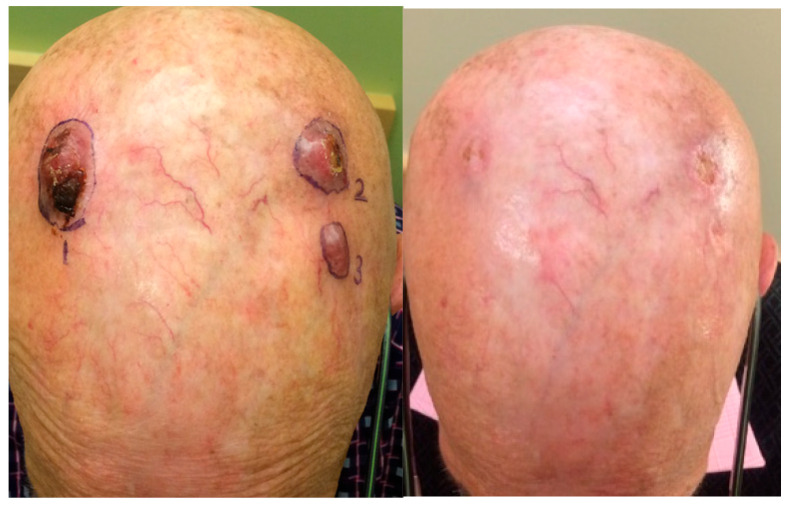
Patient with metastatic Merkel cell cancer recurring after multiple treatments (**left**). Complete regression of lesions after treatment with haNK cell infusions combined with Avelumab (**right**) [8].

NK-92 cells were also engineered to express endoplasmic reticulum (er) IL-2, which provides sufficient IL-2 intracellularly to maintain their proliferation and expansion but without significant extracellular secretion of the cytokine [4,5]. The erIL-2-engineered NK-92 cells were further modified to express high-affinity Fc-receptors (CD16_158V FcγRIIIa) called *haNK* cells (*high*-*affinity* *NK*-92) [5,6,7]. The haNK cells were part of a clinical trial at the University of Washington in patients with advanced Merkel cell cancer combined with the mAb PD-L1 Avelumab [8] [*NCT03853317*]. Two of seven patients in that study went into complete remission after having experienced various failed prior treatments [Figure 2].

Since the approval process for plasmid-engineered CAR expressing cells is less intensely regulated than for virus-based constructs, our group generated several CAR-expressing haNK cells via electroporation of plasmids. Those lines went under the designation *t-haNK* (*targeted*-*high* *affinity* *NK*) expressing CARs for CD19, CD20, Her2, EGFR, PD-L1, and CCR7 ([2,9,10,11,12] and unpublished observations). Other research groups have used lenti- or retroviruses to introduce a CAR construct into NK-92 cells, and since the cells did not have the high-affinity Fc-receptor, they went under the name taNK [13,14,15,16,17] (Table 1).

Noteworthy are the results of Wels et al. [18], which transfected NK-92 cells with a CAR for the ErbB2 (Her2) gene. In a phase I clinical trial, the cells were injected locally into glioblastoma lesions after surgery. The treatment was well tolerated, with some patients experiencing prolonged relapse-free survival [19] [NCT03383978].

Since we were unable to obtain independent funding to continue the further development of NK-92 with the goal of obtaining FDA approval for initial safety and efficacy trials, a biotech company was founded in 2002 in Chicago (*ZelleRx Inc.*, Chicago, IL, USA) that supported its further translational and clinical development. In 2010, the NK-92 assets were acquired by *Conkwest* (San Diego, CA, USA), which went public in 2014 under *Nantkwest Inc.* The preclinical and clinical development of NK-92 and its engineered variants is now with *ImmunityBio Inc.* (Culver City, CA, USA), which is headquartered in the Los Angeles area.

Since the deposit of NK-92 into cell registries, researchers worldwide have had access to these cell lines, which has resulted in a multitude of research projects and publications [2,20]. The Fc-receptor-expressing variant is particularly attractive to pharmaceutical companies for the development and activity testing of monoclonal antibodies (mAbs). To secure intellectual property rights and control the distribution of NK-92 and its variants, a subsidiary of *ImmunityBio Inc.* (Culver City, CA, USA) was created: *Brink Biologics Inc.* (Torrey Pines, CA, USA).

## 2. NK-92 as a Tool in Cancer Research

Because of its wide distribution; its relative ease of maintenance in culture; and, most importantly, because of its close similarity to human blood NK cells, researchers worldwide have generated an abundance of data with NK-92 cells and their variants. Although a few other NK-like cell lines have been identified, only NK-92 has such close characteristics to blood NK cells, has significant cytotoxic/cytostatic activity, and can be expanded under FDA-compliant conditions [2,21]. The broad range of target cytotoxicity of NK-92 is related to the fact that it expresses the full spectrum of currently known activating receptors but only very few known inhibitory receptors (LIR/ILT, CD94/NKG, KIR2DL4) [2]. The initial cell clone was established in a special culture medium containing fetal bovine serum and horse serum along with some additives [1]. We now know that the choice of culture medium can have some effect on the spectrum of receptor expression and cytotoxic activity (unpublished observation). The use of 5% human serum with X-Vivo 10 is compatible with FDA regulations for use in humans and has been and continues to be used for clinical trials.

Although NK-92 cells have broad cytotoxic and growth-inhibitory effects on cancer cell lines and primary tissue, a few cancer targets are not effectively killed by NK-92. Research is trying to determine the pathophysiology of target cell sensitivity/resistance. Some video recordings have shown that the killing of target cells occurs only when the cytotoxic granules migrate toward the synapse after NK-92 cells have made contact with the tumor where they are released [22]. However, it is still unknown what triggers the migration of the granules and the subsequent release of perforin and granzymes. What is known, though, is that targeted cell killing can be quite consistently achieved when NK-92 cells express a target-specific CAR or when they are combined with an antibody that binds the receptor to target cells. Interestingly, in contrast to perforin and granzymes, which are only released at the synapse with tumor cells, the cytokines from NK-92 cells are released across the entire cell membrane upon target cell contact [22].

## 3. NK-92 Cells Can Induce a Vaccine Effect upon Intra-Tumor Injection

**Figure 3:**
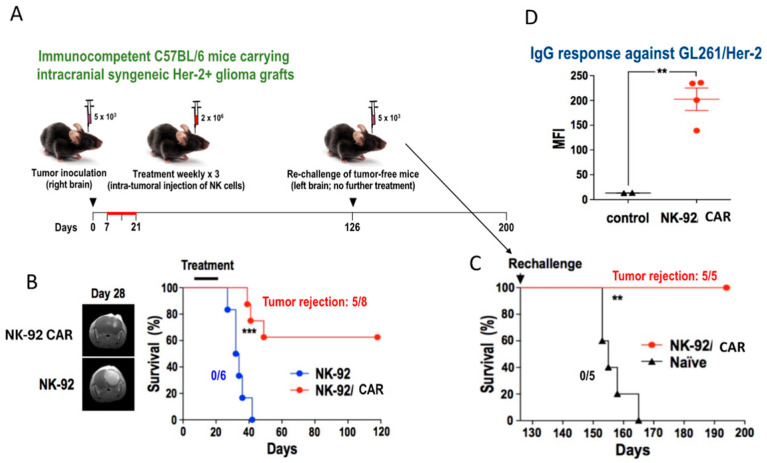
Antitumor activity of Her2 CAR NK-92 cells in immuno-competent C57BL/6 mice with stereotactically injected Her2+ glioblastoma (GL261). Seven days later, animals were treated by intra-tumor injection with 2 × 10^6^ parental NK-92 (*n* = 6) or Her-2 CAR-expressing NK-92 cells (*n* = 8) once a week for 3 weeks (red line). (**A**) Experimental setup: injections of unmodified NK-92 cells served as control. (**B**) Tumor regression as assessed by MRI on day 28 (**left**) resulting in long-term survival in 5/8 mice (**right**). (**C**) Animals that were disease-free after Her2 CAR NK-92 treatment were re-challenged on day 126 by stereotactic re-injection of GL261 cells. All mice that had received the Her2 CAR NK-92 cells survived long-term, whereas the ones that had received the unmodified (naïve) NK-92 cells died within 6 months. (**D**) IgG serum antibody levels against GL261/Her2 glioblastoma cells as determined by mean fluorescence intensity (MFI) of flow cytometry. Sera from naïve C57BL/6 mice (*n* = 2) served as control [18], MFI: Mean Fluorescence Intensity, ** *p* ≤ 0.01; *** *p* ≤ 0.001.

When Balb/C mice with A20 lymphoma were injected intra-tumor with NK-92 cells engineered to express a murine CD19 CAR, the lymphoma was eliminated in the majority of the mice [23]. When those mice were inoculated several weeks later with the same lymphoma at a different site, no tumor growth or recurrence occurred. Similar data were presented by Zhang et al. [18], who established Her2+ glioma (GL261) intra-cranially in immuno-competent C57BL/6 mice. Seven days later, the animals were treated by intra-tumor injection with either unmodified or Her2+ CAR-expressing NK-92 cells once a week for 3 weeks (Figure 3). None of the mice who received unmodified NK-92 cells had significant tumor regression, and all those mice had died by day 40 after inoculation. However, complete tumor regression occurred in 5/8 mice receiving the Her2+ CAR-expressing NK-92 cells, resulting in their long-term survival. Those mice also did not develop cancer upon the re-injection of the GL261 glioma cells on the opposite side of the brain. These results, like the ones with A20 lymphoma, strongly suggest a memory-like effect induced by CAR-targeted NK-92 cells.

## 4. NK-92 Cells for Infectious Diseases?

**Figure 4:**
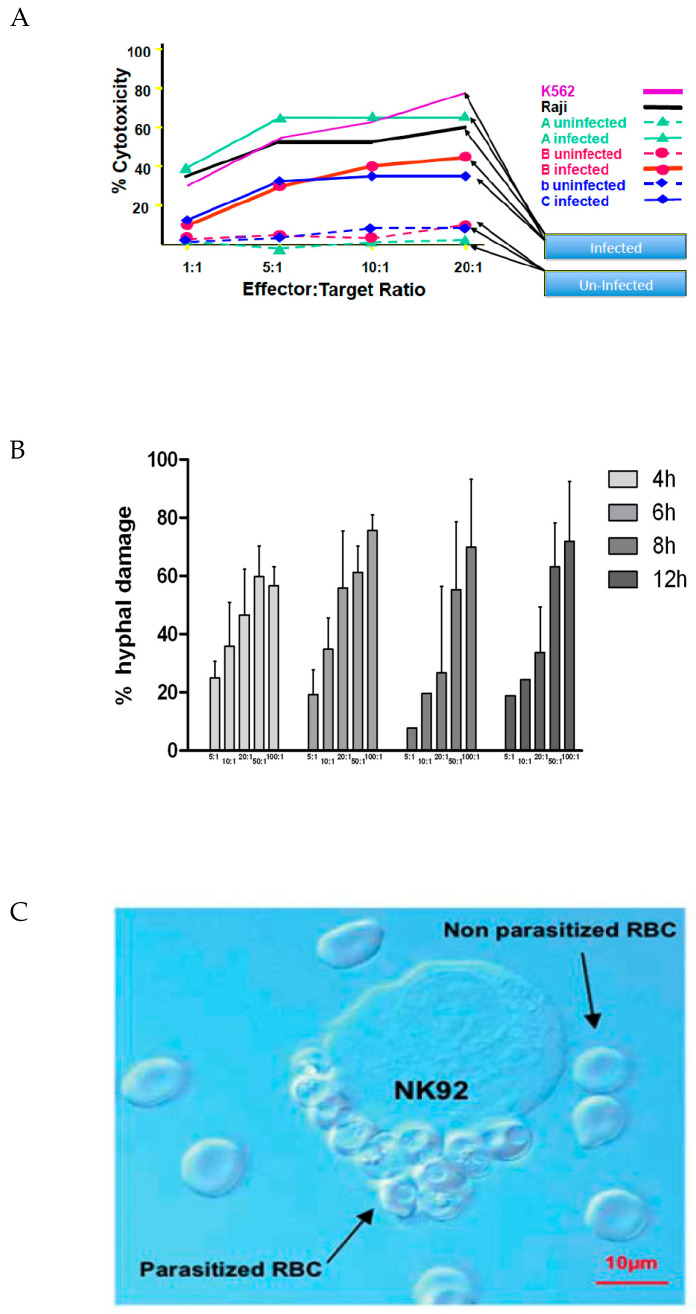
In vitro studies with NK-92 against virus, fungus, or parasite-infected cells. (**A**) PBMC infected with *Epstein*–*Barr Virus* (*EBV*) are effectively killed by a 4 h co-incubation with parental NK-92 cells, whereas non-EBV-infected PBMC are not affected. Controls with K562 and Raji targets show the (expected) cytotoxicity of NK-92 against these malignant targets. (**B**) *Aspergillus fumigatus* exposed to NK-92 cells experiencing significant hyphal damage over a 4–12 h exposure to NK-92 [25]. (**C**) NK-92 binds and kills human red blood cells (RBCs) that have been infected with *Trypanozoma cruzei* [26].

Although the focus and interest of cellular therapy has been (and still is) predominantly on patients with cancer, infectious diseases are also a significant challenge in healthcare. The recent COVID epidemic showed how important it could be to have a more universal anti-viral treatment available. NK cells are the primary and rapid immune response force in viral infections. In vitro studies with NK-92 cells have shown that they can eliminate EBV-infected lymphocytes ([24] and unpublished observation) (Figure 4A) and also effectively kill fungi (Figure 4B) [25]. NK-92 cells can also selectively bind to and kill erythrocytes that are infected with plasmodium parasites (Figure 4C) [26]. So far, however, no clinical studies in the infectious disease space have been performed with NK-92 or its variants.

## 5. Clinical Trials with NK-92 and Its Engineered Variants

**Figure 5:**
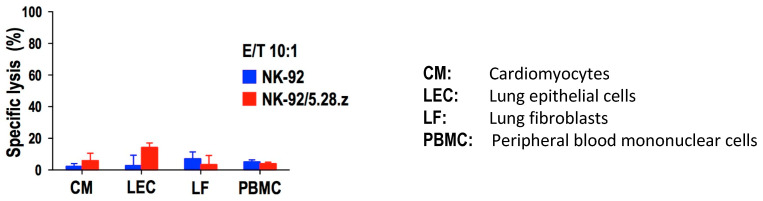
NK-92 cells and their Her-2 CAR-expressing variant NK-92/5.28.z do not display lytic activity against different human primary tissue cells, as tested in a 4 h cytotoxicity assay [16].

Before treating patients with the “original” NK-92 cells back in 2002, the FDA had to see data showing that NK-92 cells do not negatively affect healthy human cells/tissue. Independent studies confirmed that neither unmodified nor CAR-engineered NK-92 cells are cytotoxic to normal healthy tissue cells [Figure 5] [2,16].

**Figure 6:**
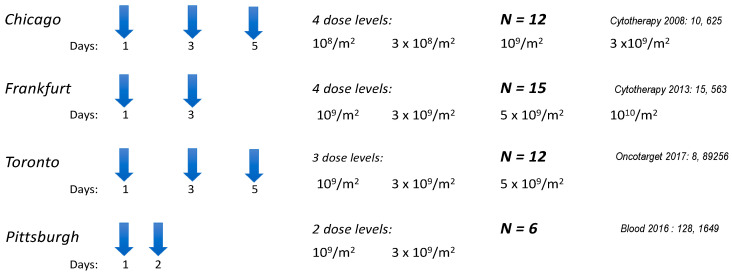
Treatment schedule of phase I studies of patients with advanced/resistant cancers with unmodified NK-92 cells [27,28,29,30].

Four phase I trials at different centers in North America and Europe enrolled 45 patients with advanced cancers treated with repeated infusions of unmodified NK-92 cells [27,28,29,30]. The infusions were given 2–3 times within one week with the rationale to infuse the cell product before a possible alloreactive effect in the patient would develop and reject the cells (Figure 6). None of the patients developed any significant side effects, and tumor responses were seen in about one-third of the treated patients (Table 2).

Noteworthy is a case report on a patient with extensive Ewing sarcoma who had failed conventional treatment but had partial remission upon NK-92 injections [31].

## 6. Engineering NK-92 with a Switch-Off Mechanism

Since the NK-92 cell line originated from a patient with lymphoma, NK-92 cells and their variants have to be irradiated (usually with 10–20 Gy) before infusion into patients, restricting their in vivo activity to about 24–36 h. To eliminate the requirement for radiation and, hence, the limited lifespan of the cells after infusion, a genetic NK-92 variant is being engineered with inducible “switch-off genes” that can be activated by inducers such as AP1903 and/or ganciclovir at any given time after infusion. These cell lines are currently being tested for their safety and efficacy in pre-clinical studies.

## 7. Making NK-92 Cells Immune–Neutral

A video recording by Eitler et al. [22] confirmed that NK-92 cells engage with multiple cancer cells over a few hours and replenish their cytotoxic molecules relatively quickly. With the goal to have NK-92 cells and their variants remain active for several days in the patient after administration, it has become relevant to make them immune-neutral even though cancer patients generally have an impaired and/or delayed immune response. In fact, earlier studies by the Toronto group had shown that an immune response to NK-92 is mitigated in cancer patients even after repeated infusions [30]. None of the 12 patients in that study developed a T-cell response, as determined by a mixed lymphocyte culture (MLC) against irradiated NK-92 cells as targets. HLA antibodies were detected in some 50% of patients but only after the second or third cycle of NK-92 cell infusions.

Moreover, the fact that the local injection of NK-92 into the tumor of fully immunocompetent mice can elicit a long-lasting memory-like response further suggests that an immediate and more pronounced anti-NK-92 immune response may not occur. Regardless, to further minimize the possibility of any rejection response, NK-92 variants have been generated that do not express HLA class I antigens [32]. To protect HLA-I-deficient NK-92 cells against lysis using allogeneic blood NK-cells via the “missing-self” pathway, HLA-E has been transduced into MHC-negative NK-92 cells to deliver an inhibitory signal through engagement with NKG2A/CD94 on PBNKs [32].

## 8. A Novel NK-92 Product: Cellular Lysate

NK-92 cells are essentially a “factory” and “cellular storage” for perforin, granzymes, and immune-active cytokines. A process has been developed that generates a cellular lysate that does not affect the activity of perforin, granzymes, or cytokines and is also GMP-compliant [33]. Studies of immunocompetent mice have confirmed that the intra-tumor injection of NK-92 lysate can eliminate cancer in over half of these animals [33]. Importantly, mice whose tumors regressed did not develop cancer upon injection of the same tumor on the opposite flank, suggesting that the NK-92 lysate also induced a memory-like effect. The advantage of NK-92 lysate is that there is no need to irradiate NK-92 cells or to be concerned about cellular rejection, making logistics for patient treatment easier than with entire cells. The lysate of NK-92 could be rendered even more active, for example, by treating them with synthetic cytokines as recently shown [34].

## 9. NK-92 Cells and Their Lysates Have Cytotoxic and Cytostatic Activities Across Species

Veterinary companies that are developing canine-specific mAbs are utilizing the NK-92 cell line and transfecting it with a canine Fc-receptor that allows them to test the ADCCs of canine mAb candidates for their target specificity and cytotoxic activity (Zoetis Inc., Personal communication). For this to be feasible, it requires that the cytotoxic molecules from the human NK-92 cell line are cross-reactive and can kill canine (xenogeneic) targets. Since human and canine cytokine genes have some 80% sequence similarity, this cross-reactivity may not be unexpected for the cellular lysate. In vitro studies have also confirmed that perforin and granzymes from NK-92 lysate are cytolytic/cytostatic to canine cancer cells [33,35], and human cytokines have already been used as part of cancer treatment protocols in dogs [36,37,38].

Natural killer cells have certain advantages over T-lymphocytes for immunotherapy, but their relatively limited numbers in blood/cord blood, a more involved expansion process, and unpredictable and variable yields have, so far, somewhat limited their development. A cell line like NK-92 that rapidly expands in cultures to large numbers can be engineered with cancer-cell-targeting receptors—made immune-neutral and with cross-species activity—and could circumvent these challenges.

## Figures and Tables

**Table 1:** Summary of past and current clinical trials with engineered NK-92 cells (taNK and t-haNK). *AML:* acute myeloid leukemia, *BCMA:* B-cell maturation antigen, *HNSCC:* head and neck squamous cell carcinoma, *MUC-1:* mucin-1, *NHL* non-Hodgkin lymphoma, *NKG2D-L* NKG2D ligands, and *TNBC* triple-negative breast cancer (clinicaltrials.gov).

NCI Trial Number	Disease	NK-92 Cell	Target Antigen	Phase	Status
NCT02742727	CD7^+^ leukemia and lymphoma	taNK	CD7	I/II	Unknown
NCT02892695	CD19^+^ leukemia and lymphoma	taNK	CD19	I/II	Unknown
NCT02944162	AML	taNK	CD33	I/II	Unknown
NCT03940833	MM	taNK	BCMA	I/II	Unknown
NCT02839954	Solid tumors	taNK	MUC-1	I/II	Unknown
NCT03383978	Glioblastoma	taNK	HER2 (ErbB2)	I	Recruiting
NCT05528341	Solid tumors	taNK	NKG2D- L	I	Recruiting
NCT03656705	Non-small cell lung cancer	NK-92 with PD-1 switch receptor	Undefined	I	Enrolling by invitation
NCT05618925	NHL	t-haNK	CD19	I	Recruiting
NCT03228667	Solid tumors	t-haNK	PD-L1	IIb	Active, not recruiting
NCT04050709	Solid tumors	t-haNK	PD-L1	I	Active, not recruiting
NCT04390399	Pancreatic cancer	t-haNK	PD-L1	II	Recruiting
NCT04847466	Gastric cancer and HNSCC	t-haNK	PD-L1	II	Recruiting
NCT04927884	TNBC	t-haNK	PD-L1	Ib/II	Terminated

**Table 2:** Outcome of 45 patients with different cancers treated in phase I studies with unmodified NK-92 cells [27,28,29,30]. CR: complete remission, PR: partial remission, MR: mixed response, SD: stable disease, HD: Hodgkin’s Disease, and HSCT: Hematopoietic Stem Cell TRansplant.

Tumor Type	Response
Melanoma	1 PR
Renal Cell Cancer	1 MR, 5 SD
Acute Myeloid Leukemia (relapsed, resistant)	1 PR
Myeloma failing HSCT	1 CR
HD failing HSCT	1 CR, 3 MR
Lung Cancer	1 MR, 2 PR
